# Supplementary figures and images for: Concatemeric Broccoli reduces mRNA stability and induces aggregates
Source: PLoS One. 2021 Aug 4;16(8):e0244166. doi: 10.1371/journal.pone.0244166 (PMC8336797; doi:10.1371/journal.pone.0244166)

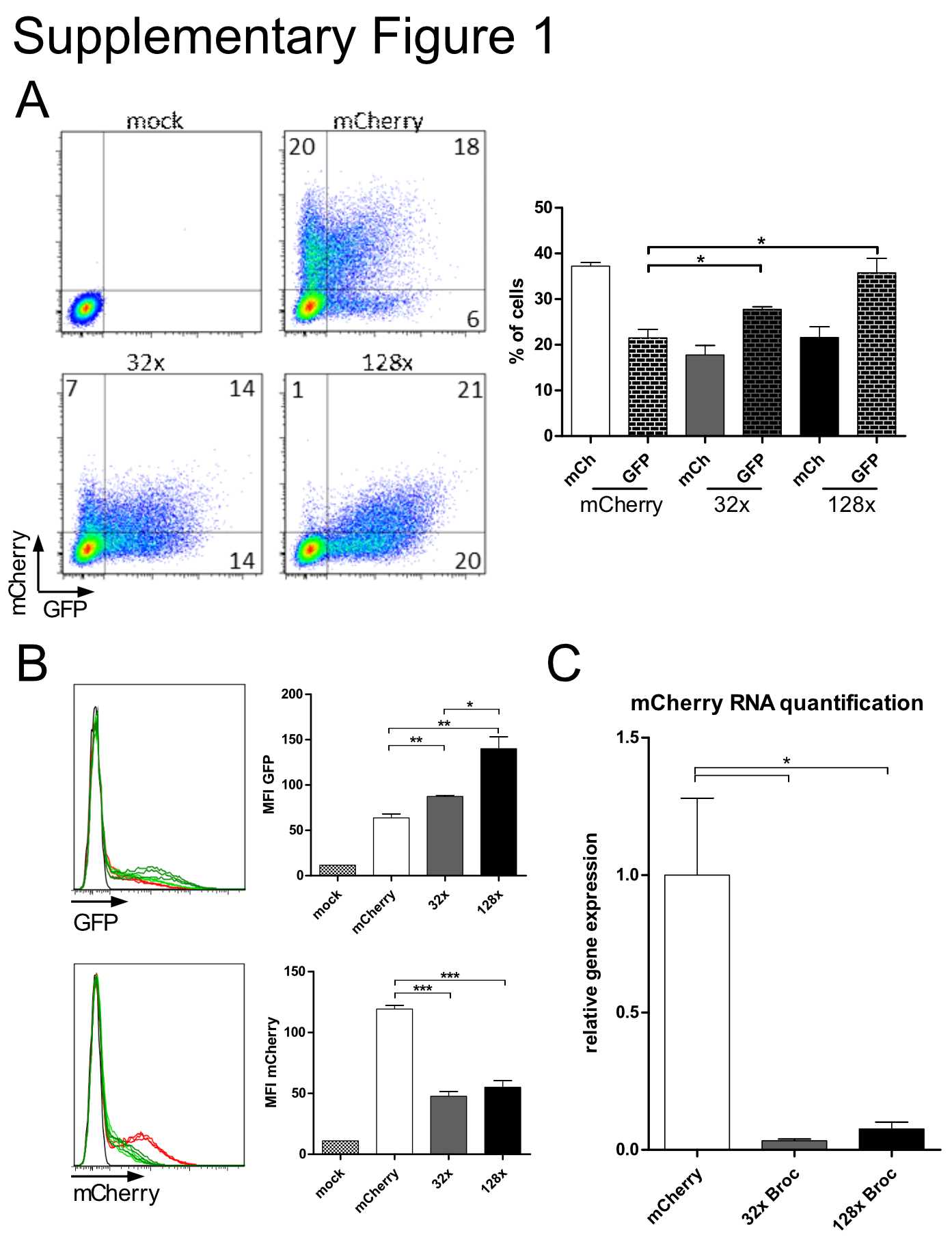

Supplement: S1 Fig — (A, B) 293T cells were co-transfected with a plasmid expressing eGFP plus mCherry, 32x, or 128xBroccoli plasmids. Percentage and intensity of eGFP and mCherry positive cells were detected by flow cytometry 24 hours later and are shown in the dot plots (A) and histograms (B), respectively. To accurately detect eGFP, DFHBI was not added to the cells. Three technical replicates are shown in the graphs. (C) The same protocol was applied and cells were collected 24 hours post-transfection for RNA isolation. RT-qPCR was performed to measure mCherry expression. The values shown were normalized to mCherry transfected cells. Statistical analysis performed by t-test. * P<0.05, ** P<0.01, *** P<0.001. (TIF) [file pone.0244166.s001.tif]

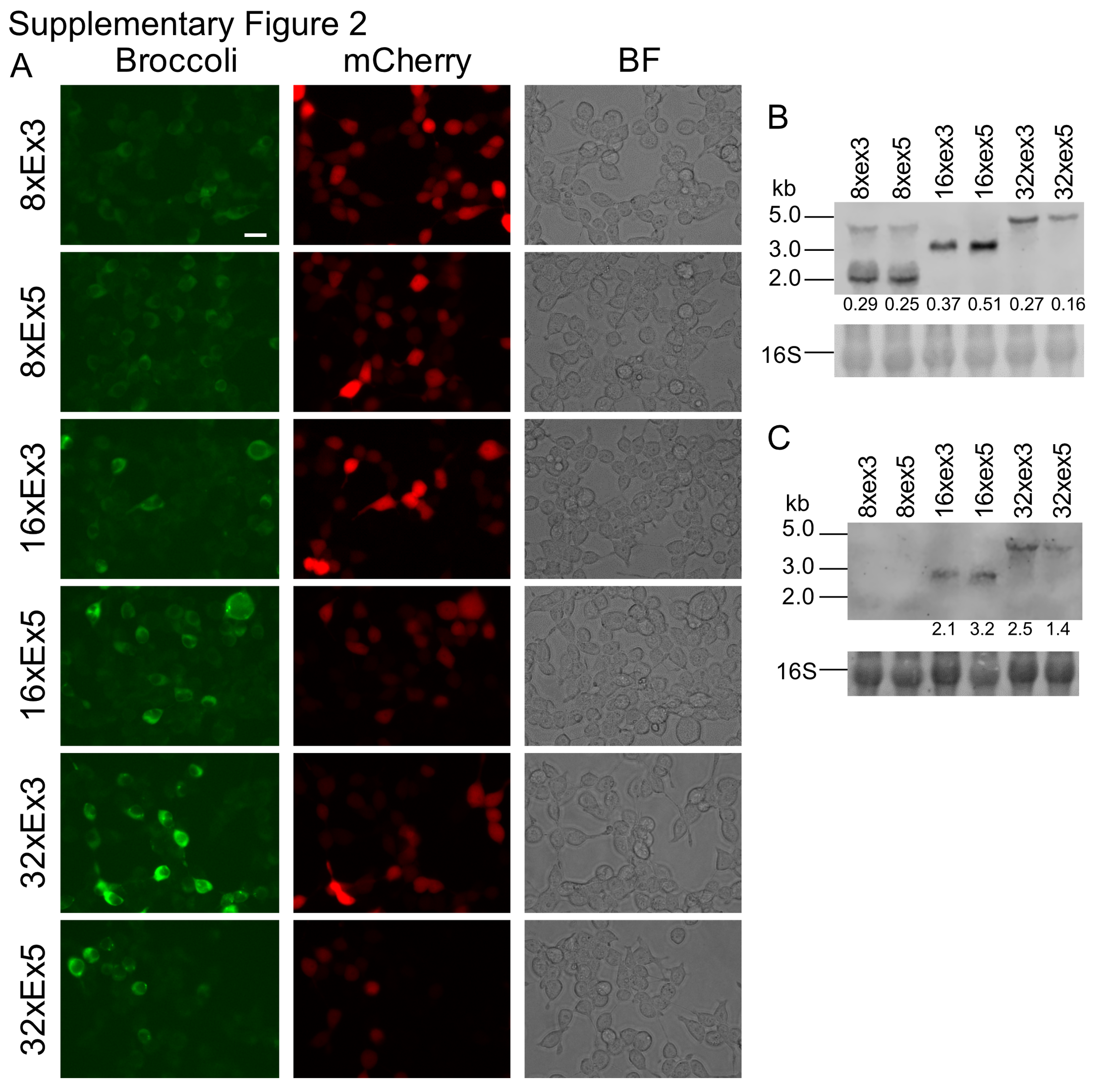

Supplement: S2 Fig — (A) 293T cells were transfected with mCherry, 8xEx3, 8xEx5, 16xEx3, 16xEx5, 32xEx3, 32xEx5, 64xEx3 Broccoli plasmids and analysed by fluorescent microscopy. (B, C) Northern blot from RNA isolated from 293T cells transfected with Broccoli plasmids containing exonic sequences. Northern membranes were probed for mCherry (B) or Broccoli (C). Values below the membranes and in the graphs indicate the intensity of the bands relative to the 16S ribosomal band for each sample and normalized to cells transfected with mCherry (B) or 4x Broccoli (C). Scale bar = 25μm. (TIF) [file pone.0244166.s002.tif]

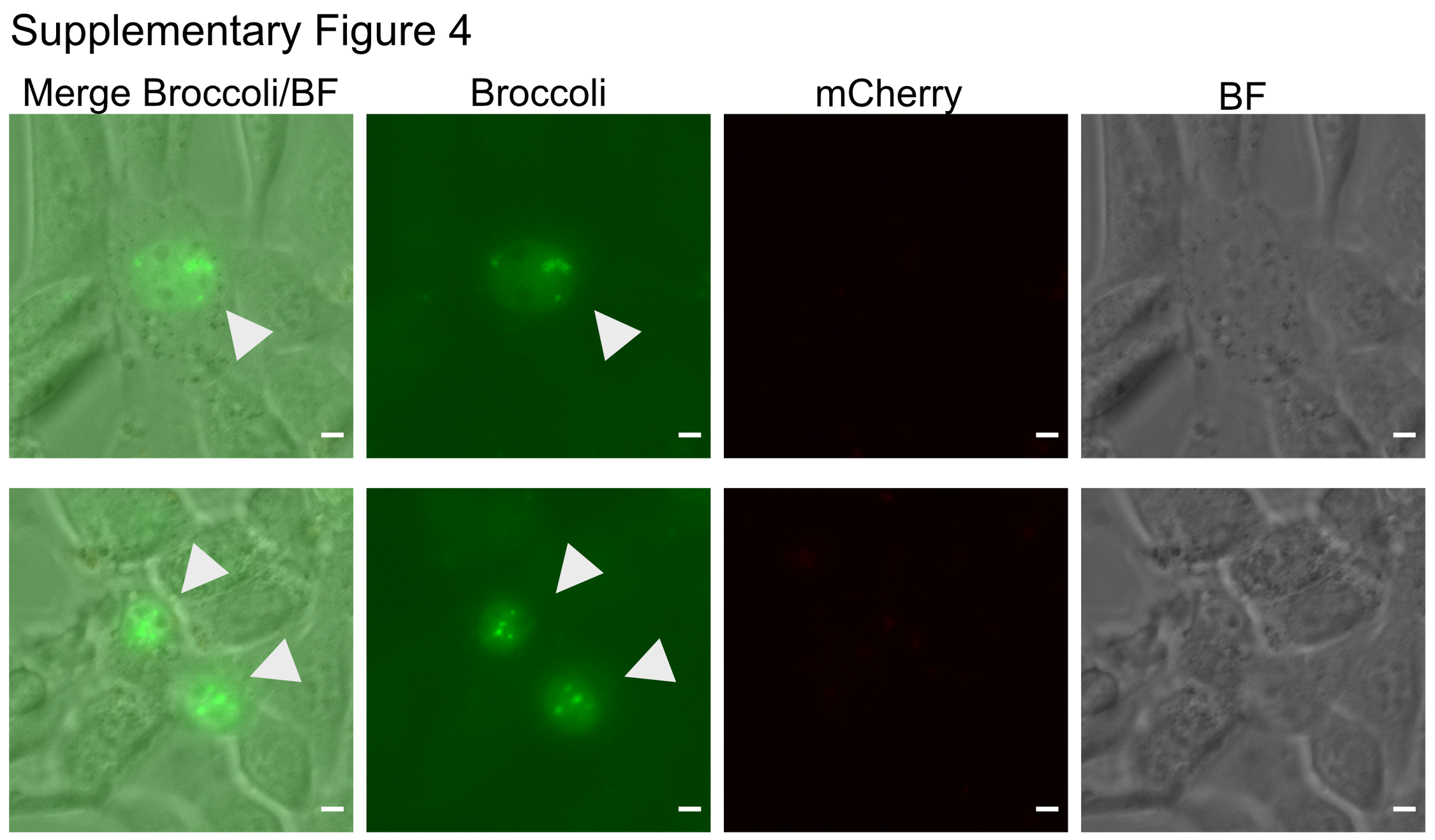

Supplement: S3 Fig — NIH-3T3 cells were transfected with 128x Broccoli and imaged by widefied microscopy 24 h later. Two representative examples are shown. Scale bar = 10 μm. (TIF) [file pone.0244166.s003.tif]

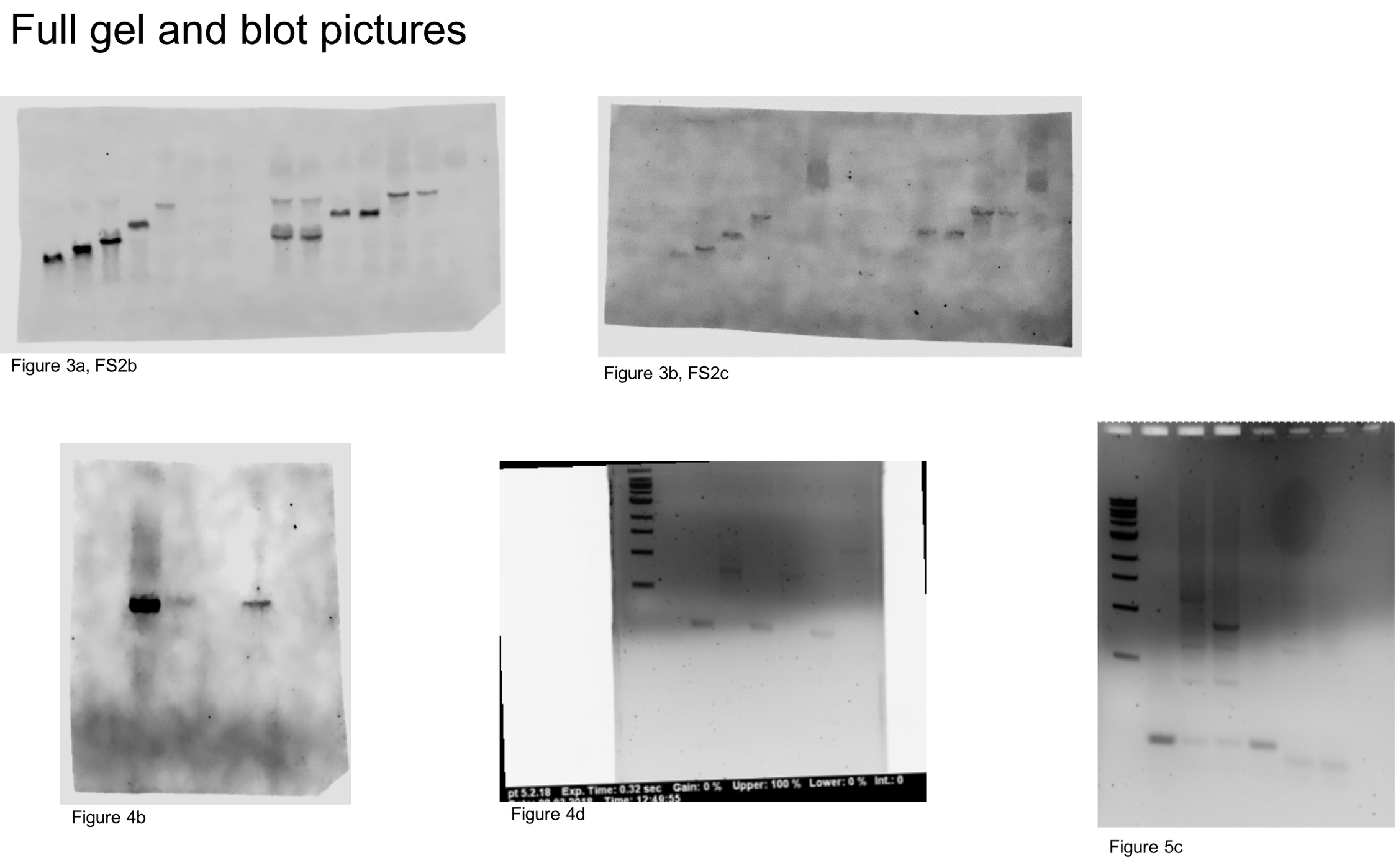

Supplement: S4 Fig — (TIF) [file pone.0244166.s004.tif]
