# Supplementary material for: Concatemeric Broccoli reduces mRNA stability and induces aggregates
Source: PLoS One. 2021 Aug 4;16(8):e0244166. doi: 10.1371/journal.pone.0244166 (PMC8336797; doi:10.1371/journal.pone.0244166)
Supplement: S1 Table — (PDF) [file pone.0244166.s005.pdf]

Primers (Supplementary Table 1):

Designation	name	sequence 5'-3'
prW83	GAPDH-rev	CTTACTCCTTGGAGGCCATGTAG
prW84	GAPDH-for	TGTCAAGCTCATTTCCTGGTATGA
prW150	Broccoli for northern	TGCCATGTGTATGTGGGAGAC
prW151	Broccoli rev northern	GAGTAAAAAGCGGACCGAAGTC
prW166	Gapdh-ex3-EcoRI-for:	GTGTGAATTCTCACCAGGGCTGCCATTTG
prW167	Gapdh-ex3-MfeI-rev	GTGTCAATTGACAAGCTTCCCATTCTCGGC
prW168	Gapdh-ex5-EcoRI-for	GTGTGAATTCCCCCTCTGGAAAGCTGTGG
prW169	Gapdh-ex5-MfeI-rev	GTGTCAATTGTAGGAACACGGAAGGCCATG
prW442	pOri-for-ApaI	GCCGCTCGAGCATGCATCTA
prW443	pOri-rev-NheI	GTGTGCTAGCGCCAGTAAGCAGTGGGTTCTC
prW459	mCherry for northern	GACATCCCCGACTACTTGAA
prW460	mCherry rev northern	TTGTAGGTGGTCTTGACCTC
prW520	5' AgeI snap-tag	CGTACCGGTATGGACAAAGAC
prW521	3' BspEI	GAGTCCGGAACCCAGCCCAGG
prW527	NheI site mChx	TGGCGACCGGTAGCGCTAGCGCCAGTAAGCAGTGGGTTCTC
prW528	ApaI site mChx	TGCAGTCGACGGTACCGCGGCCGCTCGAGCATGCATCTA
prW645	5' mCherry for	AACATCAAGTTGGACATCAC
prW646	3' Broccoli rev	GCACTGAATTCACACACTAG
prW695	3' Broccoli rev	GACACTATAGAATAGGGCCC
prW741	mCherry for	TACCGGTCGCCACCATGGT
prW742	mCherry rev	GTACAGCTCGTCCATGCCGC
prW761	mCherry for	CGGCATGGACGAGCTGTA
prW819	human c-myc for	AAAGGCCCCCAAGGTAGTTA
prW820	human c-myc rv	GCACAAGAGTTCCGTAGCTG
