# Supplementary material for: Concatemeric Broccoli reduces mRNA stability and induces aggregates
Source: PLoS One. 2021 Aug 4;16(8):e0244166. doi: 10.1371/journal.pone.0244166 (PMC8336797; doi:10.1371/journal.pone.0244166)
Supplement: S2 Table — (PDF) [file pone.0244166.s006.pdf]

Constructs in plasmids (Supplementary Table 2):

Name	Description	mCherry-Broccoli Size (bp)
mCherry	mCherry	711
4xBroccoli	mCherry-4xBroccoli	1005
8xBroccoli	mCherry-8xBroccoli	1278
16xBroccoli	mCherry-16xBroccoli	1824
32xBroccoli	mCherry-32xBroccoli	2916
64xBroccoli	mCherry-64xBroccoli	5100
128xBroccoli	mCherry-128xBroccoli	9468
8xex3 Broccoli	mCherry-8xBroccoli-exon3 GAPDH	1435
16xex3 Broccoli	mCherry-16xBroccoli-exon3 GAPDH	2138
32xex3 Broccoli	mCherry-32xBroccoli-exon3 GAPDH	3544
64xex3 Broccoli	mCherry-64xBroccoli-exon3 GAPDH	6356
8xex5 Broccoli	mCherry-8xBroccoli-exon5 GAPDH	1424
16xex5 Broccoli	mCherry-16xBroccoli-exon5 GAPDH	2116
32xex5 Broccoli	mCherry-32xBroccoli-exon5 GAPDH	3500
MCMV-mCherry	pD2 CMV strain contain mCherry	711
MCMV-16x	pD2 CMV strain contain mCh-16xBroccoli	1824
MCMV-32x	pD2 CMV strain contain mCh-32xBroccoli	2916
